# Supplementary material for: Incremental Doses of Nitrate-Rich Beetroot Juice Do Not Modify Cognitive Function and Cerebral Blood Flow in Overweight and Obese Older Adults: A 13-Week Pilot Randomised Clinical Trial
Source: Nutrients. 2022 Mar 2;14(5):1052. doi: 10.3390/nu14051052 (PMC8912345; doi:10.3390/nu14051052)
Supplement: Supplementary file 1 [file nutrients-14-01052-s001.zip › nutrients-1586171-supplementary.pdf]

Online supplementary material

Table S1: A description of cognitive tasks used during the study

Cognitive tasks	Domain	Description
**Word presentation and immediate word recall**	**Episodic memory**	At the beginning of the task battery, fifteen target words were presented on the screen, one at a time with inter-stimulus time of 1 sec. Participants were asked to remember as many words as they can. Immediately after that, a 60 second timer displayed on the screen and participants were instructed to write down as many of these words presented as they could remember within this time
**Numeric working memory**	**Working memory**	A set of five numbers were presented on the screen, separately, with an inter-stimulus time of 1 sec for the participant to hold in memory. Then, a series of numbers were displayed on the screen one at a time, and the participants were required to remember whether it had been in the original series or not and must press YES or NO as quickly as possible. This task was repeated 2 further times with different target numbers each time. Reaction time and accuracy of responses were measured.
**Choice reaction time**	**Attention**	An arrow was appeared on the screen pointing to the left or to the right. Participants were asked to press a left or right response pad button corresponding to the direction of the arrow as quickly and as accurate as possible. Fifty stimuli were presented with an inter-stimulus interval that varied randomly between 1 and 3 sec. Reaction time and accuracy were measured.
**Stroop task**	**Executive function**	A series of colour names were presented on the computer screen and there were 4 different colour inks were constantly displayed to the right of the screen. The colour ink of the colour name could be identical with the name or different. Participants were required to respond to the

Cognitive tasks	Domain	Description
		colour of the ink using the peripheral mouse and corresponding colour response buttons. 40 stimuli were presented. Task measures were accuracy, incorrect responses and reaction time.
**Digit vigilance**	**Attention**	A target digit was randomly selected and constantly presented to the right of the screen. A series of changing numbers were presented in the left of the screen and the participants were required to press the centre button whenever the two numbers are matched as quickly as possible. The task last for 3 minutes. Task measures the accuracy, reaction time and number of incorrect responses.
**Computerised corsi blocks**	**Working & spatial memory**	Nine blue identical squares were presented on the screen in random positions. A set number of those squares randomly changed to red and back to blue. Participants were asked to remember and repeat the sequence by mouse clicking on blocks. The task was repeated five times at each level of difficulty starting from 4 level (four of the nine blue squares lighting up in a random sequence). The task ended when the participant no longer correctly recall the sequence. Span score was calculated by averaging the last 3 correctly completed trials.
**Peg and ball**	**Executive function**	Two configurations of three coloured balls (blue, green, red) on three pegs were shown on the screen, one is the target configuration and one is the working configuration. The participants were required to rearrange the balls from the working configuration so that they matched the position of the balls in the target configuration using the mouse. Participants completed a total of 15 trials, containing 5 trials each that could be solved in 3, 4 and 5 moves, respectively,

Cognitive tasks	Domain	Description
		in ascending order of difficulty. Each trial generated scores for planning times prior to moving, time to complete and number of moves made above the minimum required to complete the task (errors).
**Delayed word recall**	**Episodic memory**	Participants were given 60 seconds to write down as many of the words presented at the beginning of the test. The number of words correctly recalled was recorded.
**Word recognition**	**Episodic memory**	Thirty words including the 15 original ones presented at the beginning with another 15 distractor words, were displayed one at a time randomly. The participants were asked to respond using the "yes" button on the computer keyboard if the word had been presented previously and the "no" button if it had not. The word remained on the screen until the participant responded. Mean reaction time for correct response and accuracy were recorded.
**Serial subtraction 3s & 7s**	**Working memory**	Participants were instructed to count backwords in 3s or 7s as accurately and as quickly as possible using the keyboard. A random number between 800 and 999 was displayed on the screen and it was cleared by the entry of first response. Each participant was informed verbally when they make any mistake is to carry on subtracting from the incorrect number, the subsequent responses scored as correct in relation to the new number. Number of responses, number of correct and errors responses were scored.

Table S2: Comparison of baseline characteristics of the participants who dropped out with those of the participants who completed the study

	Participants completed the study (N=50)	Participants who dropped out (N=12)	P value
Main characteristics			
Age (years)	66.2±3.8	65.8±3.7	0.78
Education (years)	15.2±2.9	16±3.6	0.41
SBP	135.9±15.0	131.8±14.1	0.68
DBP	77.1±9.7	76.2±8.8	0.42
BMI (kg/m^2)	30.5±3.7	30.0±4.1	0.76
WC (cm)	103.6±9.1	98.0±9.0	0.18
FM (kg)	32.6±8.5	30.5±9.7	0.46
FM (%)	37.9±7.7	36.9±8.5	0.68
FFM (kg)	53.1±10.0	52.1±10.9	0.74
TBW (kg)	39.2±6.8	36.7±5.1	0.23
PA (METs/wk)	3686±6062	3434±2578	0.81
Global cognitive measures			
Accuracy of attention (%)	93.8±5.9	94.8±4.1	0.56
Speed of attention (msec)	761.3±143.1	736±111.0	0.57
Accuracy of working memory (%)	93.8±8.9	96.0±4.6	0.38
Accuracy of episodic memory (%)	50.0±9.5	48.5±9.3	0.61
Speed of memory (msec)	1191.9±243.5	1152.5±260.4	0.62
Overall speed (msec)	970.9±151.3	927.6±138.7	0.37
Overall accuracy (%)	75.4±4.9	75.1±3.6	0.85
C***erebral blood flow parameters***			
Ox (%)	1.4±1.3	1.1±1.0	0.27
THb (μM/ml)	0.4±0.8	0.2±0.7	0.95
HBO (μM/ml)	0.8±0.8	0.5±0.6	0.59
HHb (μM/ml)	-0.3±0.4	-0.3±0.4	0.85

All data were analysed by independent t-test, BMI, body mass index; WC, waist circumference; FM, Fat mass; FFM, Fat free mass; SBP, systolic blood pressure; DBP, diastolic blood pressure; PA, physical activity; PA, physical activity; OX, Oxygen saturation; THb, Total haemoglobin; HBO, Oxyhaemoglobin; HHb, Deoxyhaemoglobin; G, group of intervention.

Table S3: Mean values for baseline and after 13 weeks intervention on cerebral blood flow measures assessed by qNIRS for different doses of nitrate

Measure	High NO_3^- (N=9)		Medium NO_3^- (N=13)		Low NO_3^- (N=14)		Placebo (N=13)	
	Baseline	13-weeks	Baseline	13-weeks	Baseline	13-weeks	Baseline	13-weeks
Resting CBF								
Oxygen saturation (%)	59.83±0.67	57.76±2.67	60.81±1.46	61.39±2.04	64.13±1.36	65.49±1.18	60.11±1.90	57.67±1.76
Total haemoglobin (µM/ml)	35.07±3.04	33.53±3.34	33.39±1.82	33.89±1.96	36.33±1.43	38.35±1.91	32.89±2.02	31.29±2.04
Oxyhaemoglobin (µM/ml)	21.02±1.86	19.73±2.03	20.49±1.42	21.09±1.55	23.43±1.31	25.31±1.62	20.17±1.56	18.46±1.42
Deoxyhaemoglobin (µM/ml)	14.01±1.21	13.79±1.53	12.91±0.60	12.80±0.73	12.90±0.43	13.05±0.44	12.72±0.68	12.83±0.91
Stimulated CBF								
Oxygen saturation (%)	61.69±0.26	59.72±2.56	63.11±1.42	63.39±2.02	65.52±1.42	66.70±1.23	61.66±1.86	59.77±1.51
Total haemoglobin (µM/ml)	35.07±3.00	34.02±3.45	34.02±1.80	34.35±1.99	37.10±1.42	38.37±2.03	33.41±2.00	31.88±1.96
Oxyhaemoglobin (µM/ml)	21.64±1.82	20.68±2.16	21.66±1.48	22.08±1.61	24.45±1.36	25.74±1.67	20.96±1.62	19.34±1.37
Deoxyhaemoglobin (µM/ml)	13.43±1.18	13.34±1.49	12.35±0.51	12.27±0.69	12.66±0.43	12.63±0.56	12.45±0.63	12.54±0.86

Data are presented as means ± SEM. Differences between groups were not statistically significant for any of the parameters ($p>0.05$)

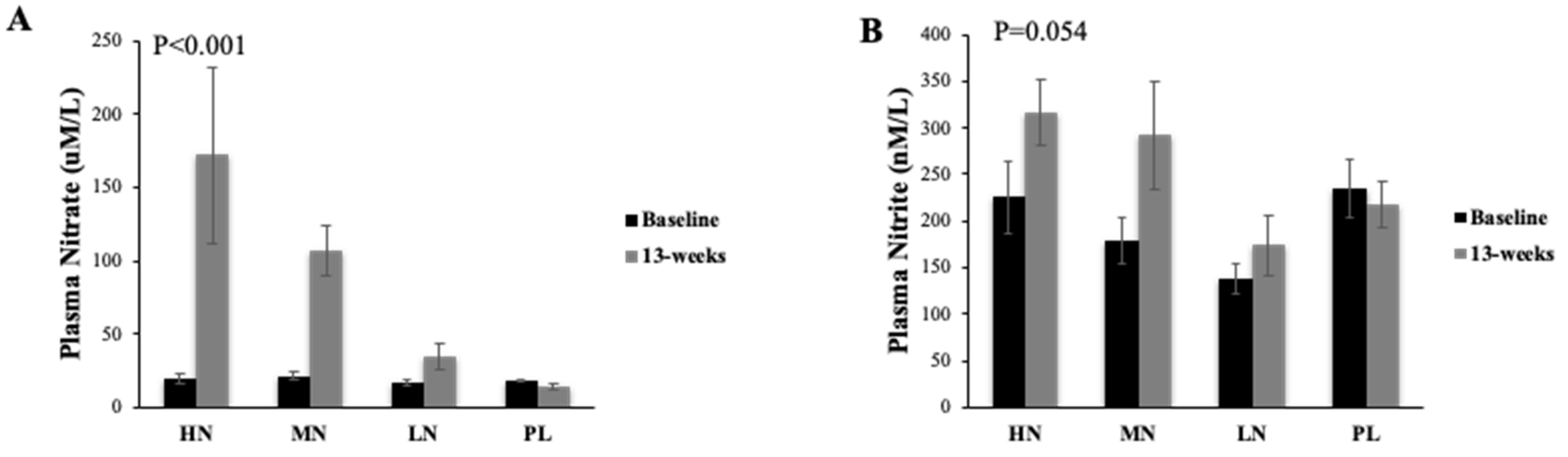

Figure S1: Mean of plasma NO_3^- (**A**) and NO_2^- (**B**) concentrations of incremental doses of dietary NO_3^- in form of BJ in older overweight and obese adults. HN (High NO_3^-; two 70 mL shots of BJ/day, morning and evening), MN (Medium NO_3^-; 70 mL of BJ/day), LN (Low NO_3^-; 70 mL of BJ every alternate days) and PL (placebo; 70 mL of NO_3^- depleted BJ). Each shot of BJ contains 400 mg of NO_3^-.P.value is for comparison of the change between different study arms. Data are expressed as mean ± standard error of the mean (SEM) (n = 49).
